# Baicalein pretreatment reduces liver ischemia/reperfusion injury via induction of autophagy in rats

**DOI:** 10.1038/srep25042

**Published:** 2016-05-06

**Authors:** Anding Liu, Liang Huang, Enshuang Guo, Renlong Li, Jiankun Yang, Anyi Li, Yan Yang, Shenpei Liu, Jifa Hu, Xiaojing Jiang, Olaf Dirsch, Uta Dahmen, Jian Sun

**Affiliations:** 1 Experimental Medicine Center, Tongji Hospital, Tongji Medical College, Huazhong University of Science and Technology, 1095 Jiefang Avenue, Wuhan 430030, China; 2 Experimental Transplantation Surgery, Department of General, Visceral and Vascular Surgery, Friedrich-Schiller-University Jena, 1 Drackendorfer straße, Jena 07747, Germany; 3 Department of Blood Transfusion, Tongji Hospital, Tongji Medical College, Huazhong University of Science and Technology, 1095 Jiefang Avenue, Wuhan 430030, China; 4 Graduate School, Southern Medical University, 1023 Shatai Nan Road, Guangzhou 510515, China; 5 Department of Infectious Diseases, Wuhan General Hospital of Guangzhou Military Command, 627 Wuluo Road, Wuhan 430070, China; 6 Animal Experiment Center, Tongji Hospital, Tongji Medical College, Huazhong University of Science and Technology, 1095 Jiefang Avenue, Wuhan 430030, China; 7 Department of Biliopancreatic Surgery, Sun Yat-sen Memorial Hospital, Sun Yat-sen University, 107 Yanjiang xi Road, Guangzhou 510120, China

## Abstract

We previously demonstrated that baicalein could protect against liver ischemia/reperfusion (I/R) injury in mice. The exact mechanism of baicalein remains poorly understood. Autophagy plays an important role in protecting against I/R injury. This study was designed to determine whether baicalein could protect against liver I/R injury via induction of autophagy in rats. Baicalein was intraperitoneally injected 1 h before warm ischemia. Pretreatment with baicalein prior to I/R insult significantly blunted I/R-induced elevations of serum aminotransferase levels and significantly improved the histological status of livers. Electron microscopy and expression of the autophagic marker LC3B-II suggested induction of autophagy after baicalein treatment. Moreover, inhibition of the baicalein-induced autophagy using 3-methyladenine (3-MA) worsened liver injury. Furthermore, baicalein treatment increased heme oxygenase (HO)-1 expression, and pharmacological inhibition of HO-1 with tin protoporphyrin IX (SnPP) abolished the baicalein-mediated autophagy and the hepatocellular protection. In primary rat hepatocytes, baicalein-induced autophagy also protected hepatocytes from hypoxia/reoxygenation injury *in vitro* and the beneficial effect was abrogated by 3-MA or Atg7 siRNA, respectively. Suppression of HO-1 activity by SnPP or HO-1 siRNA prevented the baicalein-mediated autophagy and resulted in increased hepatocellular injury. Collectively, these results suggest that baicalein prevents hepatocellular injury via induction of HO-1-mediated autophagy.

Ischemia/reperfusion (I/R) injury is a key contributing factor to liver injury, particularly following liver transplantation, liver resection, trauma, and shock. Minimizing the adverse effects of I/R injury is an important clinical problem, which could significantly reduce the incidence of acute liver failure/graft rejection and chronic liver dysfunction after transplantation^1^,^2^.

Baicalein, a main active constituent of the root of Scutellaria baicalensis Georgi, has beneficial effects in the reduction of liver injury, as recently demonstrated. *In vitro*, baicalein reduced tert-butyl hydroperoxide-induced hepatic toxicity in rat hepatocytes^3^. *In vivo*, treatment with baicalein protected animals from D-galactosamine/lipopolysaccharide (LPS) induced acute liver failure^4^, from carbon tetrachloride-induced liver injury^5^,^6^, and from concanavalin a-induced hepatitis^7^. Consistent with these findings, we recently demonstrated that baicalein ameliorated liver I/R injury in mice^8^. However, the exact mechanism of baicalein remains to be elucidated.

Autophagy is a highly conservative cellular process involving the degrading and recycling of bulk cytosolic proteins and damaged organelles to maintain cellular homeostasis^9^,^10^. A growing body of evidence indicates that autophagy is involved in several liver diseases, including toxin- and drug-induced liver damage, nonalcoholic liver disease, alcoholic liver disease, viral hepatitis, and hepatocellular carcinoma^11^,^12^,^13^. Autophagy also acts as a protective mechanism during hepatic I/R injury, and induction of autophagy has emerged as a new potential strategy to ameliorate liver function after I/R injury^14^,^15^,^16^,^17^,^18^,^19^. However, the role of autophagy in baicalein-afforded protection and the potential mechanisms involved are not fully understood.

Heme oxygenase-1 (HO-1) is a stress-inducible enzyme that shows several biological activities including anti-inflammatory, anti-apoptotic and anti-oxidant properties. HO-1 has been shown to confer protection in liver I/R injury by modulating oxidative stress and inflammation^20^,^21^. Recent studies show that in addition to these actions, HO-1 attenuates liver I/R injury by induction of autophagy^22^,^23^. However, it is unclear whether HO-1 mediated autophagy is a mechanism by which baicalein protects against liver I/R injury. Therefore, the aim of this study was to evaluate whether autophagy is an essential mediator in baicalein-afforded protection and to further explore the role of HO-1 in autophagy.

## Results

### Baicalein alleviates liver injury after hepatic I/R

**Figure 1:**
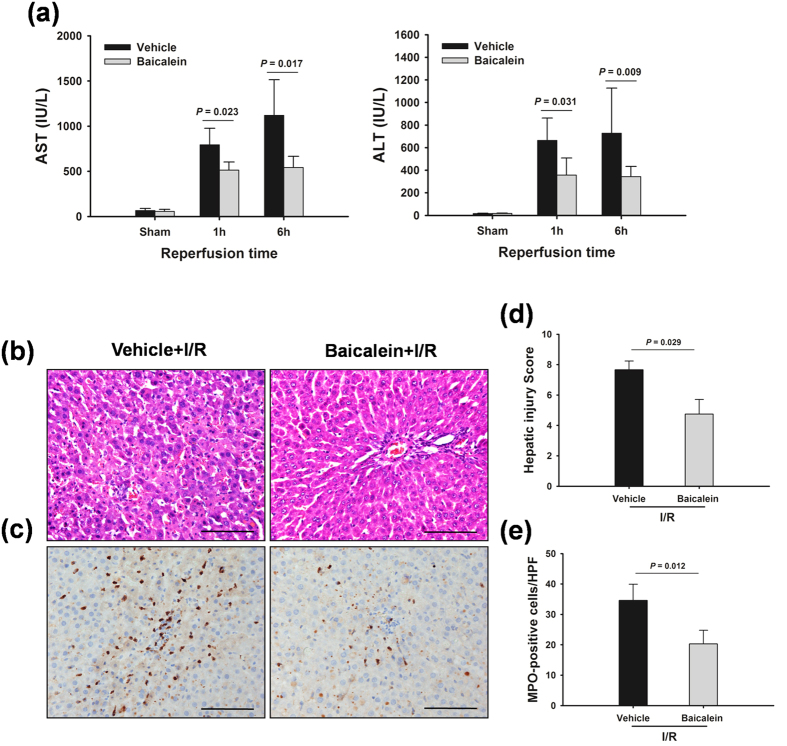
Baicalein attenuates I/R injury. Rats were pretreated with either baicalein (100 mg/kg, IP) or vehicle (DMSO) 1 h prior to ischemia. (**a**) Serum samples were collected after 1 h and 6 h reperfusion for measuring AST and ALT. Liver tissues were harvested after 6 h reperfusion, processed, and stained with hematoxylin-eosin (**b**) and MPO (**c**). Original magnification × 400, scale bars 100 μm. Representative images from 6 rats/group were selected. Damage was quantified using a semi-quantitative evaluation (**d**), and the numbers of MPO-positive neutrophils that infiltrated the livers were determined (**e**). The data are shown as the mean ± SD. n = 6 per group.

Liver damage was assessed by measuring serum aspartate aminotransferase (AST) and alanine transaminase (ALT) levels, which were markedly increased at 1 h and 6 h after I/R injury compared with those in the sham group. In contrast, treatment with baicalein prior to I/R significantly decreased the serum AST and ALT levels by 48.43% and 47.25%, respectively, at 6 h compared with the vehicle control group (Fig. 1a). These data were consistent with the histological alterations in the liver tissues. Livers from the vehicle control rats showed marked abnormalities in morphology at 6 h after reperfusion, including severe hepatocellular necrosis, cytoplasmic vacuolization of hepatocytes and neutrophil infiltration, which were dramatically reduced in the baicalein-treated rats (Fig. 1b–e).

### Baicalein induces autophagy during liver I/R injury

**Figure 2:**
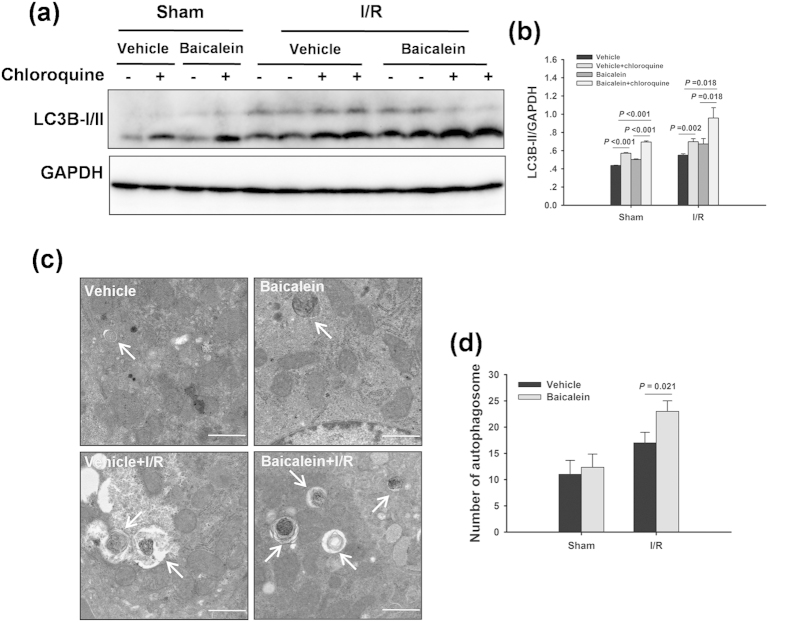
Baicalein increases autophagy after I/R. Rats were pretreated with either baicalein (100 mg/kg, IP) or vehicle (DMSO) 1 h prior to ischemia. (**a**) Western blot analysis of LC3B protein expression in the presence and absence of chloroquine (60 mg/kg). GAPDH was used as the loading control. (**b**) Densitometric analysis of LC3B-II expression. (**c**) Representative transmission electron micrographs showing autophagosomes in the ischemic lobes at 6 h of reperfusion. Autophagosomes are indicated by arrows. Scale bars 1 µm. (**d**) The numbers of autophagosomes were determined. The data are shown as the mean ± SD. n = 6 per group.

We then investigated whether baicalein pretreatment could induce autophagy in livers following I/R injury. As shown in Fig. 2a,b, the protein marker for autophagy, microtubule-associated protein 1 light chain 3B (LC3B) expression, in the livers was increased at 6 h after reperfusion in the I/R group. In contrast, baicalein treatment significantly increased the expression levels of LC3B-II in livers after I/R injury compared with those in the vehicle control group. To measure autophagic flux, a dynamic indicator of autophagic activity, LC3B-II expression levels were measured in the presence and absence of chloroquine, a specific lysosomotropic reagent that blocks lysosomal acidification and the fusion of autophagosomes and lysosomes. I/R increased autophagic flux, as indicated by a significant accumulation of LC3B-II by chloroquine. Notably, baicalein enhanced autophagic flux, as shown by the higher levels of LC3B-II in livers obtained from chloroquine-treated rats than those in the sham and ischemic livers. Furthermore, the transmission electron microscopy (TEM) analysis of the autophagosome confirmed this observation. As shown in Fig. 2c,d, an increased number of autophagosomes was detected at 6 h after reperfusion. Moreover, the number of autophagosomes was significantly increased in the baicalein-treated animals compared with the vehicle-injected animals in response to I/R injury.

### Baicalein protects against liver I/R injury via induction of autophagy

**Figure 3:**
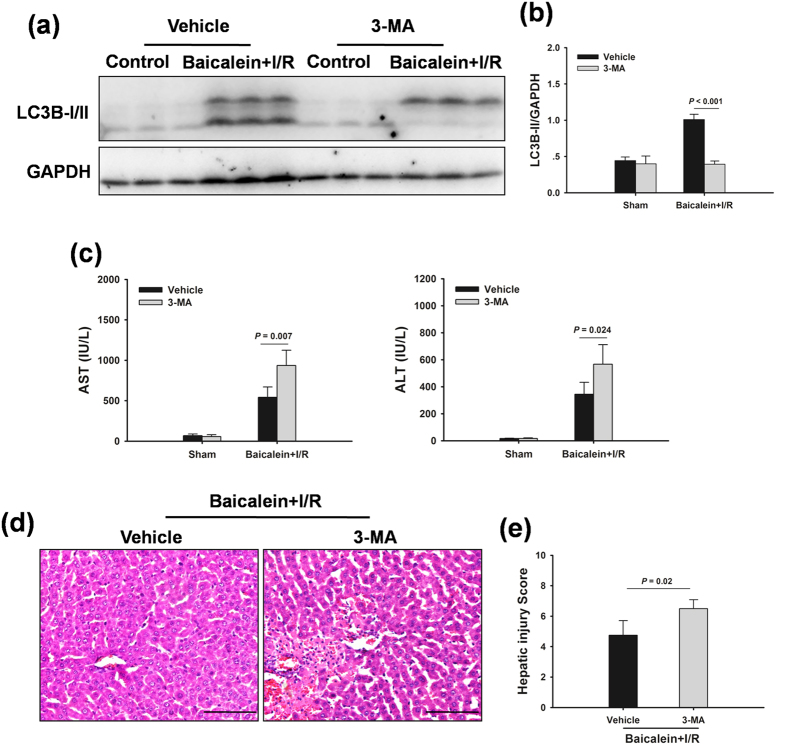
Baicalein decreases liver injury after I/R through induction of Autophagy. Rats were pretreated with 3-MA (30 mg/kg, IP) 0.5 h prior to baicalein treatment (100 mg/kg, IP) and sacrificed at 6 h after reperfusion. (**a**) Western blot analysis of LC3B protein expression in the ischemic lobes. (**b**) Densitometric analysis of LC3B-II expression. (**c**) Quantification of serum ALT and AST levels. (**d**) Representative images of liver I/R injury (Original magnification × 400, Scale bars 100 μm). (**e)** Quantification of histological damage. Data are shown as the mean ± SD, n = 6 per group.

To investigate whether the protective effect of baicalein was mediated via induction of autophagy, rats were pretreated with a pharmacological autophagy inhibitor 3-methyladenine (3-MA) prior to ischemia. As shown in Fig. 3a,b, compared with the vehicle-treated animals, 3-MA treatment resulted in a decrease in LC3B-II expression in response to liver I/R injury. Notably, the serum levels of AST and ALT were significantly increased in 3-MA-treated rats compared with those in the vehicle-treated animals (Fig. 3c). Consistently, severe histological abnormalities were present in liver tissues obtained from 3-MA-treated rats (Fig. 3d,e).

### Baicalein-induced autophagy is dependent on HO-1

**Figure 4:**
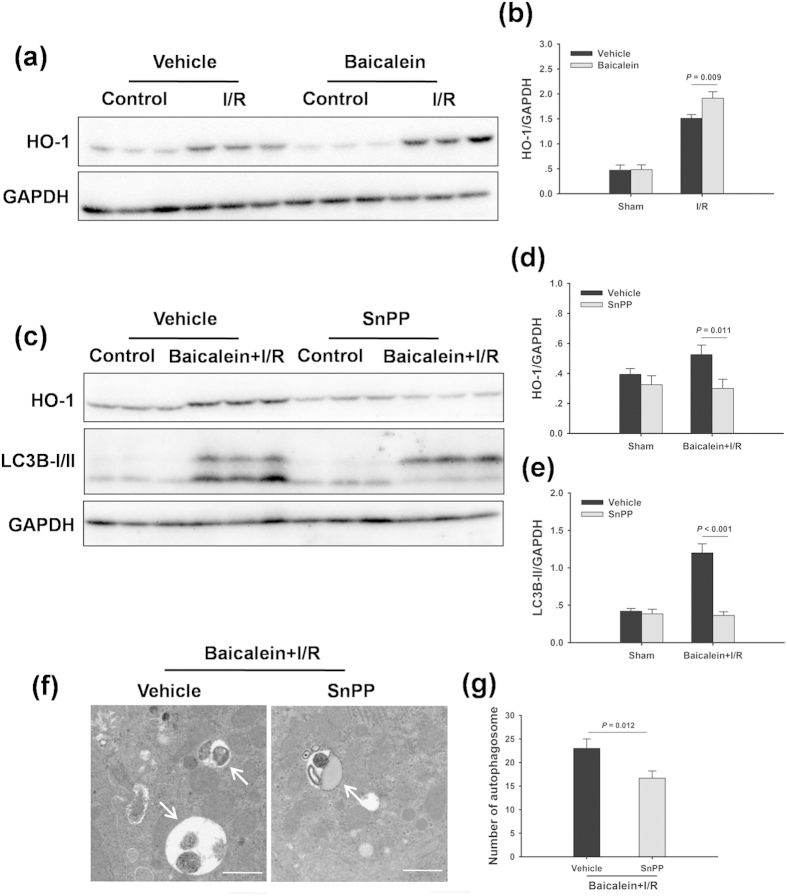
HO-1 mediates baicalein-induced autophagy. Rats were pretreated with SnPP (50 mg/kg, IP) 0.5 h prior to baicalein treatment (100 mg/kg, IP) and killed at 6 h after reperfusion. (**a**) HO-1 protein expression in the ischemic lobes was examined by western blot analysis. (**b**) Densitometric analysis of HO-1 expression. (**c**) The expression levels of HO-1 and LC3B in the ischemic lobes were examined by western blot analysis. (**d**) Densitometric analysis of HO-1 expression. (**e**) Densitometric analysis of LC3B-II expression. (**f**) Representative transmission electron micrographs showing autophagosomes in the ischemic lobes at 6 h of reperfusion. Autophagosomes are indicated by arrows. Scale bars 1 µm. (**g**) Quantification of autophagosomes. The data are shown as the mean ± SD. n = 6 per group.

**Figure 5:**
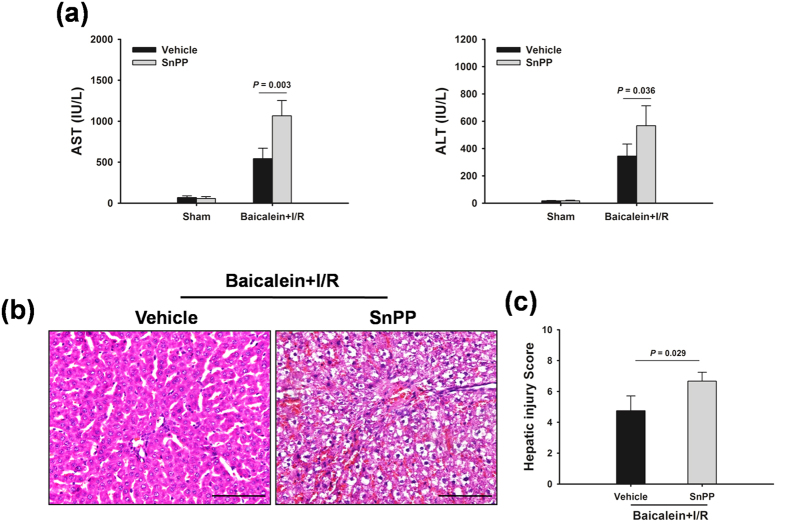
HO-1 mediates the protective effects of baicalein. Rats were pretreated with SnPP (50 mg/kg, IP) 0.5 h prior to baicalein treatment (100 mg/kg, IP) and sacrificed at 6 h after reperfusion. (**a**) Quantification of serum ALT and AST levels. (**b**) Representative images of liver I/R injury (Original magnification × 400, scale bars 100 μm). (**c)** Quantification of histological damage. Data are shown as the mean ± SD, n = 6 per group.

We next sought to identify the mechanisms underlying baicalein-induced autophagy. HO-1 is upregulated in response to I/R injury, and can reduce liver damage by induction of autophagy^22^,^23^. As shown in Fig. 4a,b, HO-1 expression was significantly increased in the livers subjected to I/R injury. In contrast, the expression levels of HO-1 in livers with baicalein were higher than those in livers with the vehicle after I/R injury. As baicalein increased both HO-1 expression and autophagy activity, baicalein-induced autophagy could result from its enhancement of HO-1. To test this possibility, HO-1 was inhibited by tin protoporphyrin IX (SnPP). Western blot analysis showed that the levels of HO-1 were decreased in SnPP-treated animals in response to liver I/R injury (Fig. 4c,d). Notably, SnPP significantly decreased the LC3B-II protein expression (Fig. 4c,e). A decreased number of autophagosomes were observed in livers from treatment with SnPP rats 6 h after reperfusion as measured by TEM (Fig. 4f,g). Furthermore, inhibition of HO-1 by SnPP significantly blocked the protective effect offered by baicalein following I/R insult. The serum levels of AST and ALT in SnPP-treated rats were higher than those in vehicle-treated animals. I/R-associated histopathologic changes were also more severe in the livers obtained from SnPP pretreatment (Fig. 5).

### Baicalein protects hepatocytes from hypoxia/reoxygenation injury via induction of autophagy *in vitro*

**Figure 6:**
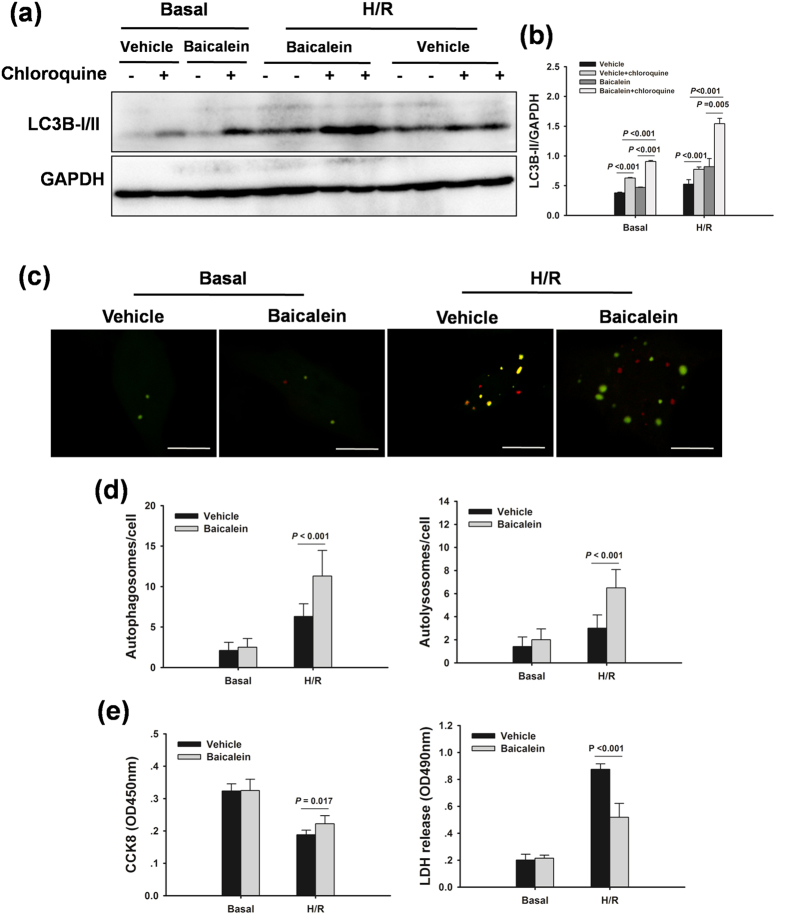
Baicalein increases autophagy in primary hepatocytes *in vitro*. Hepatocytes were treated with baicalein (5 μM) for 1 h, and followed by 6 h hypoxia and 2 h reoxygenation. (**a**) Western blot analysis of LC3B protein expression in the presence and absence of chloroquine. (**b**) Densitometric analysis of LC3B-II expression. (**c**) Hepatocytes were infected with adenovirus encoding mRFP-GFP-LC3 for 24 h, and then subjected to H/R in the presence or absence of baicalein. Representative images of fluorescent LC3 puncta are shown. The scale bar represents 30 μm. (**d**) Quantification of the number of autophagosomes represented by yellow dots in merged images and autolysosomes represented by red dots in merged images. (**e**) Quantification of cell viability and LDH release of primary hepatocytes. The experiment was performed in triplicates with similar results. The data are shown as the mean ± SD.

**Figure 7:**
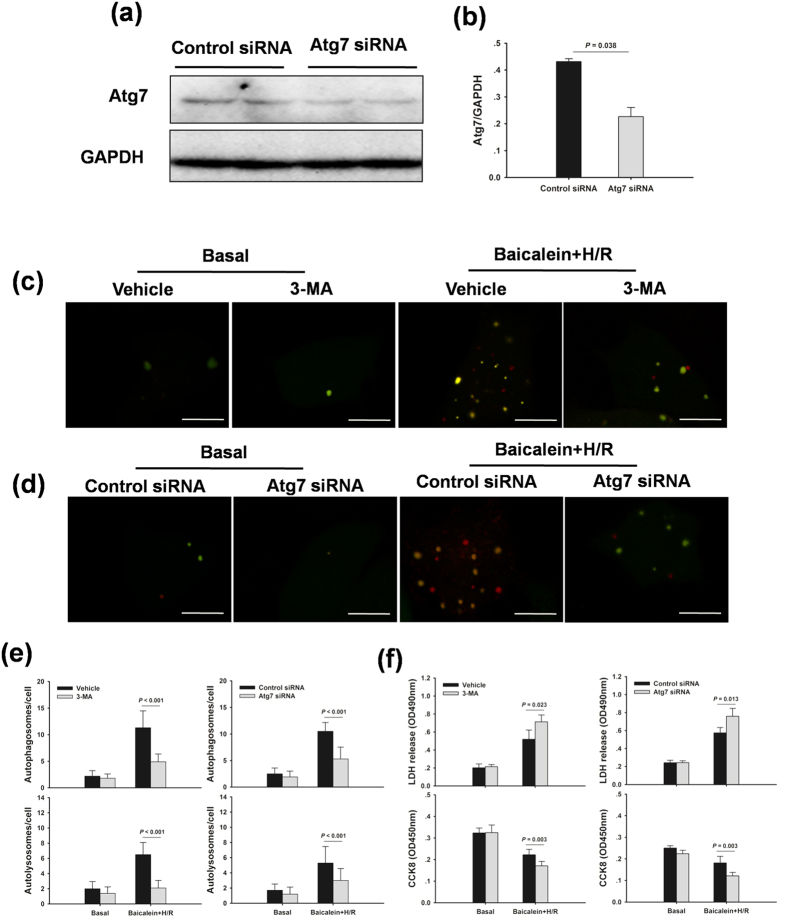
Inhibition of the baicalein-induced autophagy worsens hepatocellular injury after H/R in primary hepatocytes. Hepatocytes were pretreated with 3-MA (10 mM, 1 h) or Atg7 siRNA (50 nM, 48 h) prior to baicalein treatment (5 μM, 1 h), and followed by 6 h hypoxia and 2 h reoxygenation. (**a**) Western blot analysis of Atg7 protein expression. (**b**) Densitometric analysis. (**c**) Hepatocytes infected with adenovirus encoding mRFP-GFP-LC3 for 24 h. Representative fluorescence micrographs display autophagy vacuoles in hepatocytes with 3-MA in the presence or absence of baicalein. (**d**) Representative fluorescence micrographs display autophagy vacuoles in hepatocytes with Atg7 siRNA in the presence or absence of baicalein. The scale bar represents 30 μm. (**e**) Fluorescence micrographs of yellow (autophagosomes) and red (autolysosomes) dots were quantified. (**f**) Quantification of cell viability and LDH release of primary hepatocytes. The experiment was performed in triplicates with similar results. The data are shown as the mean ± SD.

To further determine whether autophagy could contribute to the baicalein-afforded protection against hepatocellular injury, autophagy was inhibited by 3-MA or autophagy-related protein 7 (Atg7) small interfering RNA (siRNA) in a hypoxia/reoxygenation (H/R) model *in vitro*, respectively. Consistent with the *in vivo* findings, a significant accumulation of LC3B-II by chloroquine was evident in baicalein-treated hepatocytes, indicating a robust autophagic flux with baicalein (Fig. 6a,b). In hepatocytes infected with adenovirus encoding mRFP-GFP-LC3, H/R increased the number of green and red dots. Baicalein pretreatment significantly enhances autophagic flux in cultured hepatocytes, as demonstrated by an increase in both yellow dots representing autophagosomes and red dots representing autolysosomes (Fig. 6c,d). Moreover, baicalein significantly reduced H/R-induced hepatocellular injury, as indicated by higher cell viability and lower lactic acid dehydrogenase (LDH) levels in the media than in the vehicle group (Fig. 6e). 3-MA or Atg7 siRNA-treated hepatocytes demonstrated minimal induction of autophagy compared with the vehicle-treated hepatocytes (Fig. 7c–e). Moreover, inhibition of baicalein-induced autophagy with 3-MA or Atg7 siRNA resulted in increased cell death, as measured by cell viability and media LDH levels (Fig. 7f).

### Baicalein increases autophagy via HO-1

**Figure 8:**
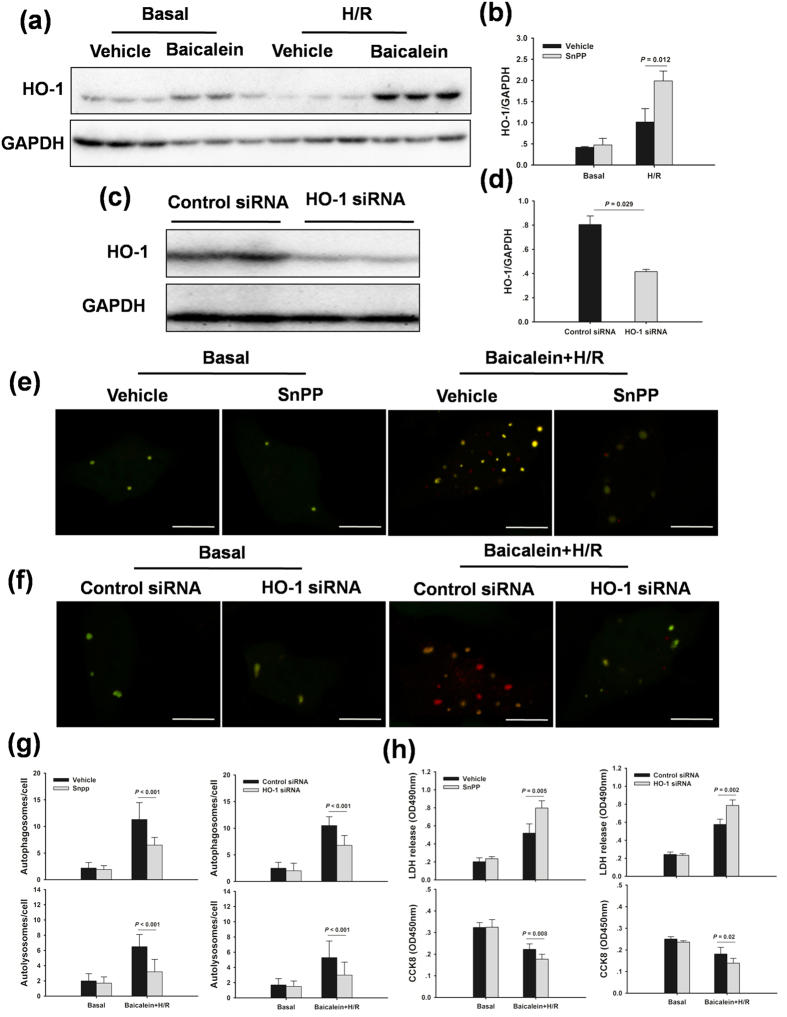
Inhibition of HO-1 prevents the baicalein-induced autophagy and protection after H/R in primary hepatocytes. Hepatocytes were treated with SnPP (50 μM, 1 h) or HO-1 siRNA (50 nM, 48 h) prior to baicalein treatment (5 μM, 1 h), and followed by 6 h hypoxia and 2 h reoxygenation. (**a**) HO-1 protein expression was examined by western blot analysis. (**b**) Densitometric analysis of HO-1 expression. (**c**) Immunoblots indicating expression of HO-1 protein after treatment with HO-1 siRNA for 48 h. (**d**) Densitometric analysis. (**e**) Representative fluorescence micrographs display autophagy vacuoles (yellow: autophagosomes; red: autolysosomes) in hepatocytes with SnPP in the presence or absence of baicalein. (**f**) Representative fluorescence micrographs display autophagy vacuoles in hepatocytes with HO-1 siRNA in the presence or absence of baicalein. The scale bar represents 30 μm. (**g**) Quantification of autophagosomes and autolysosomes. (**h**) Quantification of cell viability and LDH release of primary hepatocytes. The experiment was performed in triplicates with similar results. The data are shown as the mean ± SD.

To further investigate the role of HO-1 in baicalein-induced autophagy, HO-1 was inhibited by SnPP or HO-1 siRNA in an H/R *in vitro* model. Baicalein significantly increased HO-1 protein expression compared with the vehicle group in response to H/R (Fig. 8a,b). The number of autophagosomes and autolysosomes increased in the baicalein-treated hepatocytes, however, they were significantly decreased in hepatocytes pretreated with SnPP or HO-1 siRNA, indicating that baicalein-induced autophagy was diminished by inhibition of HO activity (Fig. 8e–g). Additionally, inhibition of HO-1 activity significantly prevented the protective effect of baicalein (Fig. 8h).

## Discussion

Recent studies have demonstrated that baicalein has a beneficial effect in liver injury caused by toxic^5^,^6^,^7^, D-galactosamine/LPS^4^ and sepsis^24^. We previously demonstrated that baicalein was also protective in a murine model of liver I/R injury^8^. However, the knowledge of the mechanism in liver I/R injury remains poorly understood. Autophagy can play a pro-survival role in liver I/R injury. Therefore, the purpose of this study was to determine whether baicalein could induce autophagy and consequently protect against liver damage following I/R injury in rats. The major novel findings of this investigation are: (1) baicalein pretreatment reduces hepatocellular injury both in I/R injury in rats and H/R injury in cultured hepatocytes; (2) the protective effect of baicalein is mediated via induction of autophagy; and (3) the baicalein-induced autophagy is dependent on HO-1.

Several previous investigations have suggested that baicalein is able to reduce I/R injury in several organs. Cui *et al.*^25^ showed that baicalein protected the brain from damage caused by permanent middle cerebral artery occlusion via down-regulation of the expression of 12/15-lipoxygenase, p38 mitogen-activated protein kinase and cytosolic phospholipase A2. Similar effects have been demonstrated in the heart. Song *et al.*^26^ reported that baicalein treatment significantly inhibited cardiomyocyte apoptosis, inflammatory responses and oxidative stress in the heart after I/R injury via modulating the activation of mitogen-activated protein kinases and Akt pathways, and suppressing the activity of nuclear factor-kappa B. The beneficial effect of baicalein was further confirmed by our previous study, which documented that baicalein could reduce liver I/R injury via inhibition of inflammation by down-regulating nuclear factor-kappa B activity, and suppression of cellular hepatic apoptosis in mice^8^. In agreement with these observations, we found that baicalein possessed strong beneficial effects against liver I/R injury in a rat model and H/R injury in cultured hepatocytes. Collectively, these observations suggest that baicalein is able to reduce I/R injury by affecting several intracellular stress and survival signaling pathways.

Autophagy has long been recognized as an adaptive response to cellular stress that avoids cell death. In the normal liver, basal autophagy performs the standard functions of degrading long-lived cytosolic proteins and damaged proteins, degrading mitochondria, regulating hepatocellular lipid metabolism, regulating immune response, and modulating cell death^11^,^12^,^13^. Autophagy is activated when the cell is subjected to metabolic stress, such as ischemia and hypoxia. Inhibition of autophagy in these conditions can lead to increased cell death. Conversely, induction of autophagy can protect animals from liver I/R injury^14^,^15^,^16^,^17^,^18^,^19^. Kim *et al.*^16^ demonstrated that autophagy was a primary catabolic process and conferred cytoprotection against prolonged liver I/R. Wang *et al.*^18^ suggested that a restoration or enhancement of autophagy might be a novel therapeutic modality to ameliorate liver function after I/R in aged livers. More recently, we demonstrated that pretreatment with lithium reduced liver I/R injury, at least in part via induction of autophagy^17^; suppression of I/R-induced autophagy by chloroquine worsened liver injury^15^, and ischemic preconditioning protected against liver I/R injury via induction of autophagy^22^. These data strongly support the view that autophagy is a protective mechanism in I/R injury. Baicalein may act on a number of stress and survival pathways, which may related to autophagy. Therefore, it is possible that the protective action of baicalein could result from its enhancement of autophagy. In the present study, we demonstrated that baicalein treatment could induce autophagy activity during I/R. To further determine the involvement of an autophagic mechanism in the protection offered by baicalein, baicalein-induced autophagy was inhibited by 3-MA prior to I/R injury. It was shown that 3-MA administration abolished the baicalein-induced protection against I/R injury. This finding was confirmed by the data from *in vivo* studies, which showed that baicalein prevented hepatocytes from H/R insult and the beneficial effect was abrogated by treatment with 3-MA or Atg7 siRNA. These investigations suggest the protective role of baicalein may be partially through induction of autophagy.

The regulatory mechanisms of autophagy during hypoxic/ischemic liver injury remain unclear. Carchman *et al.*^27^ recently reported that autophagy was mediated by HO-1 and protected against liver injury from sepsis. This finding was in agreement with our previous study, which demonstrated that ischemic preconditioning protected against liver I/R via HO-1-mediated autophagy^22^. In the current study, we found that baicalein treatment increased HO-1 expression and autophagy activity in response to I/R injury. Thus, baicalein-induced autophagy via HO-1 signaling would be necessary. Therefore, we next investigated the effect of HO-1 in baicalein-induced autophagy. The baicalein-induced HO-1 was inhibited with SnPP prior to ischemia. Furthermore, inhibition of HO-1 by SnPP prevented the baicalein-induced autophagy and attenuated the protection effect of baicalein in response to I/R injury in rats. A similar finding was observed *in vitro*; the baicalein-induced autophagy and the beneficial effect were diminished by SnPP or HO-1 siRNA, respectively. These data indicate the HO-1-mediated autophagy plays an important role in the protective effect of baicalein on liver I/R injury.

In conclusion, the present study provides evidence that baicalein could ameliorate liver I/R injury via induction of HO-1-mediated autophagy. In light of these observations, we can suggest that the therapeutic administration of baicalein might prevent liver I/R injury and autophagy is a protective molecular pathway in I/R injury.

## Methods

### Experimental design

The experiments were designed to investigate whether baicalein could protect against I/R injury via HO-1-induced autophagy in rats. To investigate whether baicalein could reduce liver I/R injury, rats were pretreated with either baicalein (100 mg/kg, intraperitoneal [IP], Sigma-Aldrich, St. Louis, MO) or vehicle (dimethyl sulfoxide, DMSO, Sigma-Aldrich) 1 h prior to warm ischemia. Rats were sacrificed at 1 h and 6 h of reperfusion. Liver injury and autophagy were analyzed. To investigate whether baicalein could protect liver I/R injury via HO-1-induced autophagy, 3-MA (30 mg/kg, IP, Sigma-Aldrich) was given to rats 0.5 h prior to ischemia to inhibit baicalein-induced autophagy. HO activity was inhibited *in vivo* through an injection of SnPP (50 mg/kg, IP, Santa Cruz Biotechnology, Santa Cruz, CA) 0.5 h prior to ischemia. To measure autophagic flux, chloroquine (60 mg/kg, IP, Sigma-Aldrich) was administered 0.5 h prior to ischemia. Rats were sacrificed at 6 h of reperfusion, and liver injury and autophagy were analyzed.

To investigate whether baicalein could protect hepatocytes from H/R injury via HO-1-induced autophagy *in vitro*, autophagy was inhibited by 3-MA (10 mM, Sigma-Aldrich) or Atg7 siRNA (50 nM, Life Technologies, Carlsbad, CA), and HO-1 was suppressed by SnPP (50 μM) or HO-1 siRNA (50 nM, Life Technologies) prior to hypoxia, respectively. For autophagic flux, hepatocytes were treated with 10 μM chloroquine. Hepatocytes were harvested after 6 h hypoxia and 2 h reoxygenation. Hepatocellular injury and autophagy were analyzed.

### Animals

Male inbred Sprague Dawley rats weighing 250–300 g were housed under standard animal care conditions and had free access to food and water. The present study was performed in accordance with the Guide for the Care and Use of Laboratory Animals prepared by the National Academy of Sciences, published by the National Institutes of Health. All animal procedures carried out in this study were reviewed, approved, and supervised by the Institutional Animal Care and Use Committee of Huazhong University of Science and Technology.

### Partial hepatic warm I/R

A partial hepatic warm I/R model was induced as described previously^28^. Briefly, rats were completely anesthetized with pentobarbital (60 mg/kg, IP). After opening the abdomen and dissecting the interlobular ligaments, all structures in the portal triad (hepatic artery, portal vein and bile duct) to the left and median liver lobes were occluded using a micro vascular clamp for 60 min. Sham control rats underwent the same protocol without vascular occlusion.

### Liver injury assessment

Blood was drawn from the postcava and centrifuged at 3,000 × g for 5 min. Serum levels of AST and ALT were determined by an automated chemical analyzer (Hitachi Co, Tokyo, Japan). For liver histopathology, liver tissues were taken from the left lobe after 6 h reperfusion and were fixed in 4.5% buffered formalin. Paraffin embedding was performed using standard techniques. Biopsies were then sectioned and stained with Hematoxylin-Eosin and were examined under light microscope for histological changes. Liver histologic injury was assessed using a semi-quantitative light microscopy evaluation^29^. Hepatic injury was demonstrated by the total score from each parameter. The presence of neutrophils in liver was assessed by myeloperoxidase (MPO) staining as previously described^8^.

### Gel electrophoresis and western blotting

Western blotting was performed as previously described^29^. Equal amounts of protein were separated on SDS-polyacrylamide gels and transferred to polyvinylidene difluoride membranes. The membranes were incubated overnight with primary rabbit anti-LC3B (1:1000, Abcam, Cambridge, UK), rabbit anti-Atg7 (1:500, Cell Signaling Technology), rabbit anti-HO-1 (1:1000, Abcam), and rabbit anti-glyceraldehyde-3-phosphate dehydrogenase (GAPDH) antibody (1:20000; Sigma-Aldrich). Blots were developed using a chemiluminescent substrate and visualized on the Kodak Image Station (Carestream Health Inc, Rochester, NY). Densitometry of bands in western blot was analyzed by Image J program (NIH, Bethesda, USA). The relative amount of each protein was normalized to the ratio of GAPDH.

### TEM analysis

TEM was performed as previously described^22^. Briefly, the liver tissues were fixed with 2.5% glutaraldehyde. Ultrathin sections were cut and doubly stained with uranyl acetate and lead citrate. Images were acquired using the Tecnai G2 electron microscope (FEI Electron Optics, Eindhoven, The Netherlands). For quantification, 10 micrographs were randomly taken for each liver sample and the total amount of autophagic vacuoles was counted.

### Primary hepatocyte isolation and culture

Primary rat hepatocytes were isolated and cultured by a modified *in situ* collagenase perfusion technique as described^30^. Briefly, after anesthetizing with pentobarbital, the liver was perfused *in situ* with warm Hank’s Balanced Salt Solution (Gibco, Gaithersburg, MD) via the portal vein and was followed by warm basic Dulbecco’s Modified Eagle’s Medium (DMEM, Gibco) containing 0.75 mg/ml collagenase D (Sigma-Aldrich) for 10–15 minutes. The liver was excised and minced in basic DMEM medium. The cell suspension was first filtered through a 70 μm nylon mesh followed by a 40 μm nylon mesh. The filtrate was centrifuged at 30 g for 5 minutes at 4 °C and the pellet was washed twice with basic DMEM medium. The viability of hepatocytes was 90% as determined by trypan blue exclusion. The hepatocytes were cultured on plates coated with rat tail collagen (Sigma-Aldrich ) in DMEM medium supplemented with 10% fetal bovine serum (10% FBS, Gibco).

### H/R model *in vitro*

To establish an H/R model *in vitro*, primary hepatocytes were placed into serum-free DMEM medium equilibrated with 1% O_2_, 5% CO_2_, and 94% N_2_. The culture plates were then placed in a hypoxia chamber (Thermo Scientific) flushed with the same hypoxic gas mixture for 6 h. The medium was then replaced with fresh medium supplemented with 10% fetal bovine serum and returned to a humidified incubator under normoxic conditions (95% air/5% CO_2_) for 2 h.

### siRNA transfection

siRNAs against rat Atg7 and HO-1 were used to inhibit Atg7 and HO-1 expression, respectively. Transfection was conducted using Lipofectamine RNAiMAX reagent (Life Technologies) following the manufacturer’s instructions. A scrambled siRNA was transfected as the negative control. After transfection for 48 h, primary hepatocytes were treated with different drugs for further analysis.

### mRFP-GFP-LC3 adenovirus transfection

Hepatocytes were infected with adenovirus encoding mRFP-GFP-LC3 (Hanbio, Shanghai, China) at a concentration of 20 multiplicity of infection. After transfection, primary hepatocytes were treated under control and experimental conditions. The cells were then fixed in 4% paraformaldehyde. Micrographs were obtained with an inverted Olympus FV1000 laser scanning confocal microscope (Olympus, Tokyo, Japan). The number of autophagosomes (red and green dots) and autolysosomes (red-only dots) per cell were calculated for evaluation of autophagic activity.

### Cell viability evaluation

A CCK-8 Cell Viability Assay Kit (Signalway Antibody, College Park, MD) was used to evaluate cell viability. Briefly, 5 × 10^3^ cells were cultured on a 96-well plate. After the desired treatment time, the medium was harvested and 100 μl CCK-8 working solution diluted with fresh medium was added into each well, and optical density (OD) was measured at 450 nm after incubating for 2 h using a multi-mode micro plate reader (BioTek, Winooski, VT). Meanwhile, LDH levels in the cultured medium were assessed by a colorimetric LDH cytotoxicity assay (Biovison, Milpitas, CA) according to the manufacturer’s protocol. OD was measured at 490 nm with a reference wavelength at 630 nm.

### Statistical analysis

The data were expressed as the means ± SD. Differences between groups were evaluated for significance using a one-way ANOVA combined with a Bonferroni’s post hoc test. All tests were performed using SigmaStat v3.5 (Systat-Software, Erkrath, Germany). A p-value below 0.05 was considered to indicate statistical significance.

## Additional Information

**How to cite this article**: Liu, A. *et al.* Baicalein pretreatment reduces liver ischemia/reperfusion injury via induction of autophagy in rats. *Sci. Rep.* **6**, 25042; doi: 10.1038/srep25042 (2016).
